# Responsiveness of cats (*Felidae*) to silver vine (*Actinidia polygama*), Tatarian honeysuckle (*Lonicera tatarica*), valerian (*Valeriana officinalis*) and catnip (*Nepeta cataria*)

**DOI:** 10.1186/s12917-017-0987-6

**Published:** 2017-03-16

**Authors:** Sebastiaan Bol, Jana Caspers, Lauren Buckingham, Gail Denise Anderson-Shelton, Carrie Ridgway, C. A. Tony Buffington, Stefan Schulz, Evelien M. Bunnik

**Affiliations:** 1 Cowboy Cat Ranch, Mico, TX 78056 USA; 2 Institute of Organic Chemistry, Technische Universität Braunschweig, Hagenring 30, 38106 Braunschweig, Germany; 3 Big Cat Rescue, 12802 Easy Street, Tampa, FL 33625 USA; 4 Room 8 Memorial Cat Foundation, 8354 63rd Street, Riverside, CA 92509 USA; 5 Mary S. Roberts Pet Adoption Center, 6165 Industrial Street, Riverside, CA 92504 USA; 6 School of Veterinary Medicine, University of California Davis, 1 Garrod Drive, Davis, CA 95616 USA

**Keywords:** Olfaction, Plants, Behavior, Tigers, Pheromones, Nepetalactone, Actinidine, Iridomyrmecin, Isodihydronepetalactone

## Abstract

**Background:**

Olfactory stimulation is an often overlooked method of environmental enrichment for cats in captivity. The best known example of olfactory enrichment is the use of catnip, a plant that can cause an apparently euphoric reaction in domestic cats and most of the *Pantherinae*. It has long been known that some domestic cats and most tigers do not respond to catnip. Although many anecdotes exist of other plants with similar effects, data are lacking about the number of cats that respond to these plants, and if cats that do not respond to catnip respond to any of them. Furthermore, much is still unknown about which chemicals in these plants cause this response.

**Methods:**

We tested catnip, silver vine, Tatarian honeysuckle and valerian root on 100 domestic cats and observed their response. Each cat was offered all four plant materials and a control, multiple times. Catnip and silver vine also were offered to nine tigers. The plant materials were analyzed by gas chromatography coupled with mass spectrometry to quantify concentrations of compounds believed to exert stimulating effects on cats.

**Results:**

Nearly all domestic cats responded positively to olfactory enrichment. In agreement with previous studies, one out of every three cats did not respond to catnip. Almost 80% of the domestic cats responded to silver vine and about 50% to Tatarian honeysuckle and valerian root. Although cats predominantly responded to fruit galls of the silver vine plant, some also responded positively to its wood. Of the cats that did not respond to catnip, almost 75% did respond to silver vine and about one out of three to Tatarian honeysuckle. Unlike domestic cats, tigers were either not interested in silver vine or responded disapprovingly. The amount of nepetalactone was highest in catnip and only present at marginal levels in the other plants. Silver vine contained the highest concentrations of all other compounds tested.

**Conclusions:**

Olfactory enrichment for cats may have great potential. Silver vine powder from dried fruit galls and catnip were most popular among domestic cats. Silver vine and Tatarian honeysuckle appear to be good alternatives to catnip for domestic cats that do not respond to catnip.

**Electronic supplementary material:**

The online version of this article (doi:10.1186/s12917-017-0987-6) contains supplementary material, which is available to authorized users.

## Background

The living environment of domestic cats (*Felis catus*) largely determines their quality of life. Cats kept indoors exclusively can become bored and stressed more easily than cats that can roam around freely outside, especially in the absence of good (feline or human) company. Lack of environmental features such as vertical space, hiding places, scratching posts, and opportunity to play and chase are known to contribute to variable combinations of behavioral problems (e.g., aggression, destructive behavior and excessive grooming) and physical disease such as lower urinary tract disease, upper respiratory disease, inappetence, and obesity [1]. These problems compromise feline health and well-being, and also contribute to cats being relinquished or returned to shelters [2, 3]. Although effective improvements to the living environments of cats are well known and often used to increase their quality of life, olfactory stimulation is often overlooked as a means of enrichment, despite recognition of the importance of environmental smells for cats by the American Association of Feline Practitioners and the International Society of Feline Medicine [4].

Cats have highly developed olfactory systems and are capable of detecting volatile stimuli (odorants) as well as pheromones. Furthermore, some plants are known to produce semiochemicals that attract and stimulate cats. This serendipitous effect is the result of the plant releasing allomones into the air to repel herbivorous insects that (may) cause damage to the plant, or to attract predators and parasitoids for these insects. The chemical profiles of these phytodistress signals may differ between plants, the attacking herbivore, and whether or not the plant is being attacked [5]. One of these plants is catnip, which produces allomones that happen to have an apparently euphoric effect on cats, including the big cats [6, 7]. This effect is quite rare in animals, and could be used to improve the quality of cats’ lives [8]. The effect of catnip is caused exclusively by its smell rather than its taste [6]. Typically, cats respond to catnip by sniffing, licking and biting it, shaking their head, rubbing their head, chin or cheeks against it, and rolling over, sometimes accompanied by drooling and kicking the material with their hind feet [6, 9–11]. This behavioral response is not sexual, [10, 11] and catnip is generally believed to be neither addicting nor harmful [12]. Unfortunately, approximately one out of every three domestic cats does not respond to catnip [9–11]. Furthermore, whereas lions, jaguars, leopards and snow leopards appear to be sensitive to catnip, most tigers do not respond to it [6, 7].

Other plants can have similar positive effects on cats (reviewed by Tucker and Tucker [13]). In 1906, a letter was published in Science about cats appreciating silver vine as a new “cat delicacy” after it was imported from China by the arboretum of Harvard University [14]. This plant, also known as matatabi in East Asia, is commonly used as a cat stimulant in Japan. Similar anecdotes of plants attracting and exciting cats exist for, among others, Tatarian honeysuckle, valerian root, and the fresh roots of Indian nettle (*Acalypha indica*) [15]. Unfortunately, to the knowledge of the authors, none of these effects have been documented.

To evaluate whether veterinarians and veterinary technicians were familiar with catnip alternatives, we asked 38 veterinarians and 6 veterinary technicians in the USA and Australia if they were aware of silver vine or Tatarian honeysuckle. Twelve were feline-only veterinarians and six out of six were feline-only veterinary technicians. Thirty-six of the 38 veterinarians (95%) and all six veterinary technicians (100%) answered this question negatively, suggesting that most veterinarians and veterinary technicians (in these countries) are unaware of silver vine or Tatarian honeysuckle and their effects on cats.

**Fig. 1:**
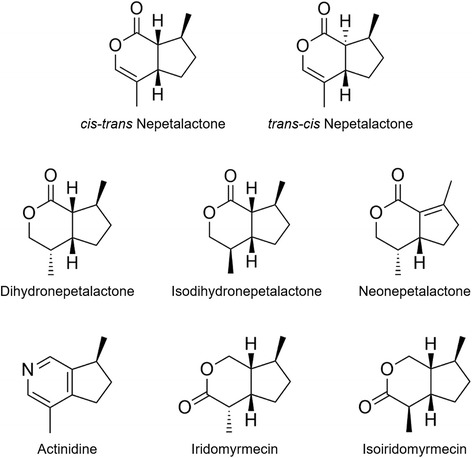
Compounds identified in catnip and silver vine known or believed to have a stimulating effect on cats

Nepetalactone has been identified as the compound causing the catnip effect [16]. Interestingly, not one, but half a dozen other active components similar in structure to nepetalactone (Fig. 1) have been identified in silver vine [6, 17–19], one of which also was detected in valerian root [20] and two, recently, in Indian nettle [15]. To the knowledge of the authors, nothing is known about the active compound(s) present in Tatarian honeysuckle. Data on the percentage of cats responding to silver vine are scarce and of poor quality [21] and, to the knowledge of the authors, non-existent for Tatarian honeysuckle and valerian root.

The purpose of this study was to compare the responsiveness of domestic cats to catnip and to these catnip alternatives. In addition, we investigated if the pattern of responsiveness to these plants could be explained by the presence or absence of certain chemical compounds.

## Methods

### Study population

One hundred randomly selected cats of six months or older living in a cat sanctuary (Room 8 Memorial Cat Foundation (R8MCF), Riverside, CA, USA) (*n =* 62), in an adoption guarantee (formerly referred to as no-kill) shelter (Mary S. Roberts Pet Adoption Center (MSRPAC), Riverside, CA, USA) (*n =* 20), in homes (Riverside, CA, USA) (*n =* 12), and in an American Association of Feline Practitioners-certified cat friendly practice with gold status (Riverside Cat Hospital, Riverside, CA, USA) (*n =* 6) were included in this study. The R8MCF housed cats in five rooms, with on average 20–25 cats per room (averaging two meters squared by two and a half meters high per cat), whereas the MSRPAC housed cats in individual cages (averaging three meters squared by two meters high per cat). About 55% of the surface space at the R8CMF was outdoors, and the cats could freely move between indoors and outdoors. Cats at the MSRPAC were allowed outside of their cages into a 100 square meters open room to interact with each other daily for eight hours. All cats at both facilities had continuous access to hiding places, cat beds, shelves and cat trees, scratching boards, litter boxes and water. The cats at the R8CMF had continuous access to dry food and received wet food at least once daily, whereas the cats at the MSRPAC received dry food twice daily. At the R8CMF window fans were used to help cool the rooms, whereas at the MSRPAC a constant temperature of 24 °C was maintained.

Cats in isolation areas or with signs of upper respiratory disease were excluded. Data were missing for some cats because they were adopted while the study was in progress. Only cats for which we had data for all four stimuli (*n =* 95) were included in the response pattern analyses. Cat age, sex, and breed were recorded when available. Ages were available for 89 cats, although for seven senior cats it could only be reliably determined they were at least 10 years of age. To test whether responsiveness to the plant materials differed between younger, generally more playful, cats and older cats, the group of 89 cats was divided into a younger (≤4 years and 10 months, *n =* 45) and older (>4 years and 10 months, *n =* 44) group using the median age of these 89 cats as a cutoff between the groups. The average age for the younger group was 1 year and 11 months (range 7 months to 4 years and 10 months) and at least 9 years and 2 months (range 5 to 16 years) for the older group. Cats also were classified (assisted by staff and volunteers who helped socialize the cats) into three different behavioral categories: (i) scared or shy (defined as avoiding humans by hiding or not leaving their location after humans entered their room), (ii) intermediate (defined as showing an interest in human presence and enjoying being petted when approached by humans), and (iii) affectionate or friendly (defined as immediately approaching humans entering their room, wanting to be petted) to study the influence of social behavior on the response to the plant materials.

### Plant materials

Dried, cut and sifted organic catnip (*Nepeta cataria*) leaves and flowers were obtained from Frontier (Norway, IA, USA) and Smarty Kat (San Rafael, CA, USA). Dried, cut and sifted organic valerian (*Valeriana officinalis*) root was purchased from Organic Bio Herbs (Woodland Park, NJ, USA) and Frontier (Norway, IA, USA). Tatarian honeysuckle (*Lonicera tatarica*) wood and sawdust were purchased from The Cat House Inc. (Calgary, AB, Canada). Powder from dried silver vine (*Actinidia polygama*) fruit galls was purchased from Smack (Nagoya, Japan) and Gendai Pharmaceutical (Tokyo, Japan). Dried normal silver vine fruit and silver vine fruit galls were kindly provided by Hangzhou Botanical Technology (Hangzhou, China). We were unable to obtain any Indian nettle material for this study. To prevent possible degradation of active compounds, all plant materials were ordered just prior to the start of the experiments. During the study period (early March till the middle of April in 2016) all plant materials were stored tightly sealed in their original bag, at room temperature and in the dark.

### Exposure

**Fig. 2:**
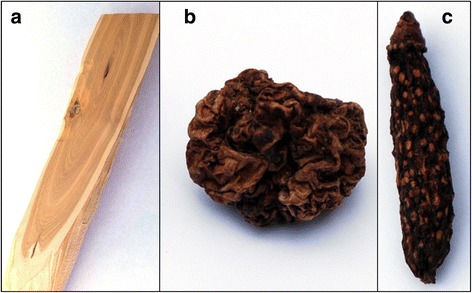
Photographs of several of the plant materials used in this study. **a** Tatarian honeysuckle wood (10 cm long, weighing 15 g). **b** A dried silver vine fruit gall, also referred to as deformed fruit (2 cm long, weighing 1.5 g). **c** A dried normal silver vine fruit (2.5 cm long, weighing 0.5 g)

Cats were exposed to 0.5 to 1.0 g silver vine powder (corresponding to 1–2 packets as sold by the manufacturers) inside a thin, porous sock (sock hereinafter), or 0.5 to 1.0 g silver vine powder spread out on approximately 500 square cm of a piece of 0.5 square m of Frieze carpet (carpet hereinafter). This type of carpet was used because it allowed more direct exposure to the materials than did the socks, while still minimizing the ability of the cats to ingest the plant material. Equal volumes of catnip (5 g), valerian root (15 g) and Tatarian honeysuckle sawdust (15 g) were offered inside a sock. This amount of plant material filled the sock about half full, and allowed tying a knot to close off the sock. Tatarian honeysuckle also was offered as an approximately 10 cm long piece of wood (Fig. 2a). Catnip (5 g) and valerian root (15 g) also were offered on carpet. We chose these amounts and volumes of catnip, Tatarian honeysuckle and valerian root to ensure that the cats would be exposed to an amount of active compounds that would allow for a positive response. We did not use a similar volume of silver vine powder because this would greatly exceed the manufacturer’s recommendation. Furthermore, the high price and limited supply of commercially available silver vine powder outside of East Asia would prevent the use of such large quantities in everyday life (e.g., cat owners, shelters). Results from preliminary experiments demonstrated that 1 g of silver vine powder was enough to elicit a positive response in each of the cats tested (Additional file 1). Socks or carpet were placed on the floor of the cat’s environment, within one meter of the cat’s head, in its line of sight or in the center of the room, respectively. When a cat seemed unaware of the presence of the plant material, the sock was repositioned within sight of the cat, once. Plant materials were never forced onto any of the cats, nor were they repositioned when a cat walked away from it. Cats responding positively to the plant materials on the carpet also responded positively to the plant materials in the sock (personal observation), suggesting that the fabric did not significantly interfere with the exposure of the active compound(s) to the cats. All plant materials were stored inside separate, sealed plastic bags to prevent cross-contamination. Identical carpet without plant materials and an identical, empty sock were used as negative controls.

**Fig. 3:**
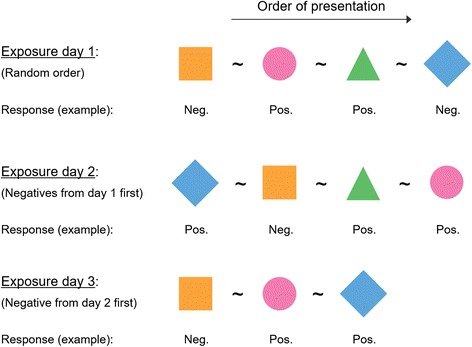
Schematic overview of a typical exposure experiment for the domestic cats. This experiment was done for each cat. The different shapes (*square, circle, triangle* and *diamond*) represent the various plant materials the cats were exposed to. A negative control (not shown in the figure) was always offered together with each plant material. The wash-out period is represented by a tilde. The responses are an example; these are not results. On the first exposure day *circle* and *triangle* were removed from the cat as soon as a positive response was scored. This was done to prevent the cat from not responding to *diamond* because of loss of interest, fatigue or saturation to active compounds that also may be present in the plant materials the cat had already been exposed to during the experiment on this day. *Square* and *diamond* were offered first (in random order) on the second day the cat was tested, because no positive response was observed on the previous testing day. This was done for the same reasons as previously mentioned. *Diamond* was removed when the cat was scored positive for this sample. *Triangle* and *circle* were offered to the cat last (in random order), to try to confirm the results obtained on the first test day, and also to establish that the cat’s environment (e.g., noises, other cats present) and its physical and mental state were not preventing the cat from responding positively. This method was repeated on day 3: the sample to which the cat did not respond positively (*square*) was offered first, followed by samples to which the cat had already responded positively. In this example the cat would have been scored negative for *square* and positive for *circle*, *triangle* and *diamond*. Pos., positive; Neg., negative

The majority of the cats were exposed to all four plant materials on three different days (2 to 16 days apart), at different times of the day (morning, afternoon or evening), for up to one hour. The minimum number of exposures for each cat was two, on different days, and the minimum exposure duration for each cat was ten minutes per exposure. All cats were exposed to the plant materials presented in a sock and the majority of them also were exposed to a piece of Tatarian honeysuckle wood, and once to the plant materials on the carpet. Each plant material was offered at separate times, in random order, with a washout period of at least five minutes in between to avoid recording false positive responses resulting from a carry-over effect. The length of this washout period was based on results from preliminary experiments (Additional file 1). New plant material was only offered to a cat when it would not or no longer show any of the behavior associated with the catnip response (see below) when offered a negative control sock. When a positive, complete response (described below) was recorded, the stimulus was removed from the cat. This was done to prevent possible response saturation (e.g., because an active compound may be present in more than one plant material or because the cat became fatigued) from negatively affecting responses to the plant materials tested subsequently. In addition, plant materials to which the cat responded positively were offered last during subsequent exposures on other days. Observing a positive response after a negative one demonstrated that environmental (e.g., distractions), mental (e.g., fear) and physical (e.g., disease) conditions did not prevent the cat from responding positively. This too was done to prevent scoring false negative responses. All cats were exposed to the plant materials in their normal living environment, and only after a period of at least ten minutes during which the cats could acclimate to the presence of the investigator. Each cat was given the opportunity to experience the plant materials without interference or intimidation from other cats and in the absence of other possible distractions, which sometimes required retesting cats at other moments. A schematic overview of a typical exposure experiment is presented as Fig 3.

### Scoring

Ninety-eight of the 100 cats were scored live by the same researcher (SB), who knew which plant materials were being offered to the cats at each exposure. Two cats living in a private home were scored (by SB) based on video recordings. Cats showing the characteristic catnip response (sniffing, licking, shaking their head, rubbing (chin/cheek) and rolling on their back, sometimes accompanied by drooling and raking (Table 1)) to a plant material were scored as positive for that plant material. Each positive response was scored as either intense/complete (i.e., sniffing and licking, as well as chin/cheek rubbing or rolling over, for >10 s) or mild/partial (i.e., sniffing and licking only [9] for >15 s non-stop, or an intense/complete response for <10 s). Cats’ responses were scored as negative when none of at least two exposures resulted in a positive response to the plant material. Negative responses of cats present in the same room but not exposed were not recorded.

**Table 1:** Ethogram of domestic cat and bobcat behavior [6, 39] observed during a positive response to catnip, silver vine, Tatarian honeysuckle or valerian root

Behavior	Description
Sniffing	The cat smells the plant material by inhaling air through its nose.
Licking	The cats tongue protrudes from its mouth and strokes the plant material.
Shaking head	The cat rotates its head from side to side with the plant material in its mouth.
Rubbing (chin)	The cat rubs its chin against the plant material.
Rubbing (cheek)	The cat rubs its cheek against the plant material.
Rolling on back	The cat rolls onto its back, with its belly exposed and all paws in the air.
Raking ^a^	The cat makes kicking movements with one or both hind legs against the plant material held by the cat, usually with both fore paws.
Drooling	The cat produces excess saliva that is visible outside of the cat’s mouth.
Undulating skin (back)	Wavelike motion of the cat’s skin in the dorsal lumbosacral region as the underlying cutaneous trunci muscles rhythmically contract and relax. Associated with arousal during play. Not to be confused with feline hyperesthesia syndrome.

^a^ This behavior is also referred to as hind leg kicking or bunny kicking

### Exposure to various parts of silver vine

To study the response of cats to various parts of the silver vine plant in more detail, nine randomly selected domestic cats (three males and six females, aged 8 to 60 months; three domestic medium haired and six domestic short haired) at the MRSPAC were exposed on multiple days to the following six plant materials, all contained within socks: 20 g dried silver vine fruit galls, 20 g dried normal silver vine fruit, 1.5 g powder of dried normal silver vine fruit, sun-dried and fresh leaves (2 and 5 g, respectively) from a three year old silver vine plant not bearing fruit galls (Forestfarm Nursery, Williams, OR, USA) and 15 g woodchips made from commercially available silver vine wood sticks (Duobi Pet Supplies, Hangzhou, China) (Table 2). To generate the powder, two gram of dried normal silver vine fruit was ground using a Mixer Mill 400 (Retsch, Newtown, PA, USA) at 25 cycles per second for three minutes. In all experiments, contact time between the cat’s face and the sock was recorded. The plant material was removed after five minutes of indifference, or after the cat stopped actively engaging with the sock for one minute.

**Table 2:** Overview of the experiments in which cats were exposed to different parts of the silver vine plant

Exposure	Silver vine material	Response	
		Negative	Positive
1	Normal fruit	9	0
	Galled fruit	1	8
2	Normal fruit	8	0
	Normal fruit (powder)	8	0
3 & 4	Control	8	0
	Woodchips	7	1
	Dried leaves	8	0
	Fresh leaves	8	0
5 & 6	Woodchips ^a^	0	1
	Galled fruit	0	1

^a^ The sock with the woodchips was offered first; the sock with the fruit galls was added after the cat lost interest in the sock with the woodchips. During all other exposures socks containing the silver vine plant material were offered simultaneously to the cats

### Other *Felidae*: tigers and bobcats

Nine tigers (*Panthera tigris*) (seven females and two males, age 16 to 20 years, with age unknown for three of them) and five bobcats (*Lynx rufus*) (three females and two males, age 8 to 23 years) living at Big Cat Rescue, a sanctuary for exotic cats, in Tampa, FL, USA were included in this study. Twenty gram of catnip (Frontier) or 1.5 g of silver vine powder (Smack) was offered to the tigers inside a folded paper bag (50 × 47 × 30 cm) with small holes made in it (*n =* 6) or uncontained (*n =* 3) by a Big Cat Rescue staff member (LB) who was certified to hand out enrichment to cats of all sizes. Tigers were first offered catnip, followed by silver vine five minutes later. If a tiger responded positively to the catnip, silver vine was offered at least 5 min after the tiger had lost interest in the catnip. In the absence of a positive response to catnip, silver vine was offered to the tigers without first removing the catnip, which, in some cases, resulted in exposing them to the silver vine within a couple of meters of the catnip. All bobcats received catnip (*n =* 1) or silver vine powder (*n =* 4) in crumpled up paper bags (13 × 8 × 25 cm) with no holes made in them. The plant materials were placed within 0.5 meter of the cat’s face, without disturbing them, so that all animals were aware of the presence of the materials. Minimum possible exposure to each plant material was five minutes for each cat. The cats were not tested between 11:00 and 16:00, because of their low level of activity during these hours.

### Chemical analysis

Dried catnip (leaves and flowers), dried normal silver vine fruit, dried silver vine fruit galls, dried valerian root and Tatarian honeysuckle wood, taken from the batches used for the responsiveness studies, were analyzed using gas chromatography coupled with mass spectrometry to determine concentrations of five known or claimed cat-stimulating compounds isolated from catnip and silver vine: *cis-trans* nepetalactone [12, 16, 22, 23] (CAS Registry Number 21651-62-7), *trans-cis* nepetalactone [16, 22, 23] (17257-15-7), actinidine [17, 18] (524-03-8), iridomyrmecin [17–19] (485-43-8) and isodihydronepetalactone [19] (17672-96-7). For extractions, 0.5 g of each plant material was extracted with 3 ml of dichloromethane while stirred at room temperature for 7 days. The solutions were filtered through a short silica gel column and concentrated by evaporating the solvent at room temperature to 1.5 ml. One hundred μl tridecyl acetate (1.001 g/l) was added to each sample as internal standard to allow for quantification of the compounds and comparison between samples. Extracts were analyzed by gas chromatography coupled with a mass selective detector (GC-MS, GC 7890A/MSD 5975C, Agilent Technologies, Santa Clara, CA, USA). The GC-MS system was equipped with an HP-5 ms fused silica capillary column (30 m, 0.22 mm internal diameter, 0.25 μm film (Agilent Technologies)). Conditions were as follows: inlet pressure: 67 kPa, He-flow: 1.2 ml/min, injector: 250 °C, transfer line 300 °C, electron energy 70 eV. The GC oven temperature was kept at 50 °C for 5 min, followed by raising the temperature with 5 °C/min to 320 °C. Identification of compounds was performed by comparison of their mass spectra and retention indices (determined from a homologous series on n-alkanes (C8-C32)) to those of reference compounds (nepetalactone) and commercial mass spectral libraries (actinidine, iridomyrmecin and isodihydronepetalactone) (Wiley 7, NIST 08). The amount of compound is expressed per gram of dried plant material. The lower limit of detection under these conditions was 0.2 μg per gram.

### Statistical analysis

Statistical analysis was performed using GraphPad Prism version 7.02 (GraphPad Software Inc., La Jolla, CA, USA). A *P*-value < 0.05 was considered statistically significant. Overlaps between responses to the various plant materials were calculated and plotted as a Venn diagram in R (version 2.7.0) using a custom script from Dr. Thomas Girke (University of California, Riverside, CA, USA).

## Results

**Fig. 4:**
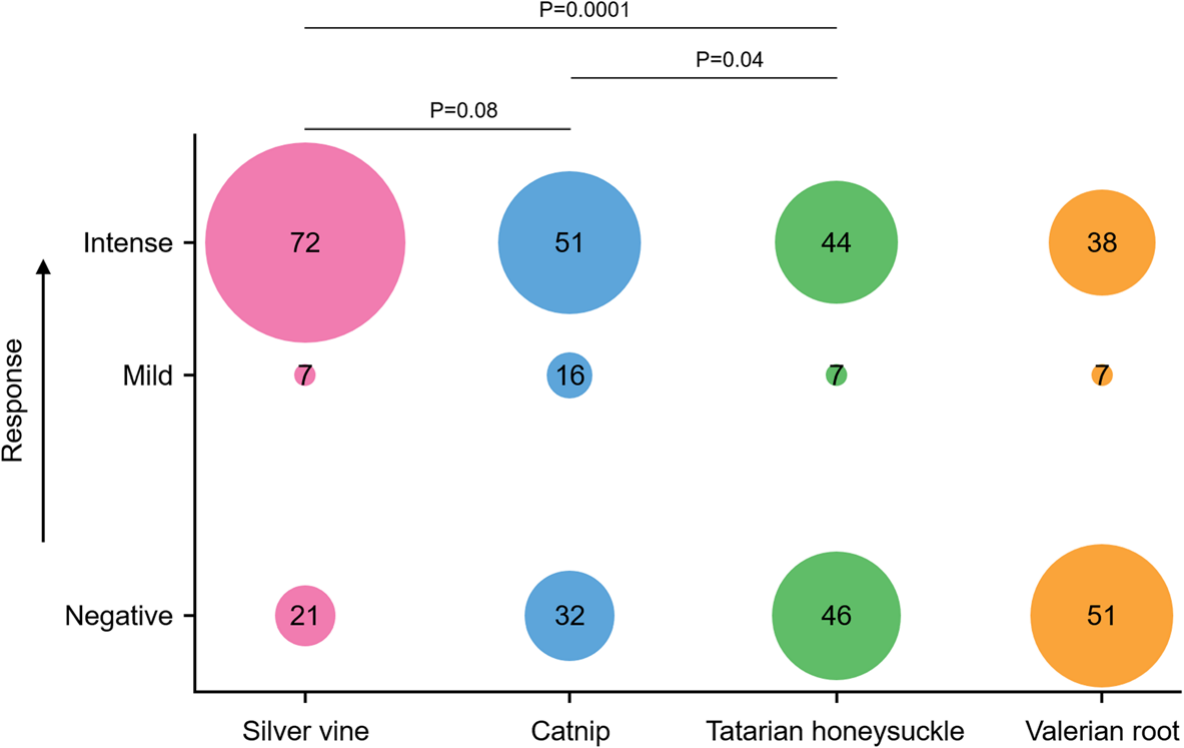
The number of cats that responded to silver vine, catnip, Tatarian honeysuckle and valerian root. The response of the total study population to each plant material is shown in a different color. Significantly more cats responded positively to silver vine and catnip than to Tatarian honeysuckle and valerian root. Responses to silver vine were more intense than to catnip (*P =* 0.02)

To investigate the responsiveness of domestic cats to catnip and catnip alternatives, we exposed cats to catnip, silver vine, Tatarian honeysuckle and valerian root in their normal living environment (see Methods). None of the cats studied responded positively to the control sock or carpet. In agreement with the results from our preliminary experiment (Additional file 1) we did not observe positive responses to the control sock after removing the plant materials from the cat, suggesting no or a minimal carry-over effect. No differences in response to the plant materials offered inside a sock or on carpet were observed, therefore these results were not recorded separately. Seventy-nine of the 100 domestic cats responded positively to silver vine (Fig. 4 and Additional file 2). Significantly more cats responded to silver vine (79%) (*P =* 0.0001, Fisher’s exact test) or catnip (68%) (*P =* 0.04) than to Tatarian honeysuckle (53%) or valerian root (47%) (Fig. 4). Although no statistically significant difference was observed between the percentage of cats responding to silver vine or catnip (*P =* 0.08), the response to silver vine was more intense than to catnip (*P =* 0.02, Fisher’s exact test).

Our study population consisted of 35 neutered male cats, 55 neutered female cats and 10 neutered cats of unknown sex. No differences were observed between the number of male and female cats responding to the four plant materials (Fisher’s exact test, Additional file 2). In addition, the average number of stimuli that the cats responded to was similar for males (2.6 out of 4, *n =* 32) and females (2.4 out of 4, *n =* 53), and no difference in response intensity was observed between male and female cats (Fisher’s exact test).

We found no evidence that younger age was associated with increased responsiveness. There was no difference in the number of cats responding positively to the various plant materials between the younger (*n =* 45) and older (*n =* 44) groups of cats (Fisher’s exact test, Additional file 2) and cats in the younger group did not respond to more plant materials than did cats in the older group (2.3 (*n =* 43) and 2.8 (*n =* 42) out of 4, respectively). However, more partial/mild responses and less complete/intense responses to catnip were observed in the older group as compared to the younger group (12% mild and 88% intense in the younger group compared to 35% mild and 65% intense in the older group, *P =* 0.04, Fisher’s exact test). No differences in response intensity were observed for the other three plant materials between the younger and older group (Additional file 2).

We did not find an association between responsiveness and behavioral category. Scared or shy cats responded as often and as intensely to the various plant materials as did affectionate or friendly cats (Chi-square test, Additional file 2). Cats categorized as either scared/shy (*n =* 36), intermediate (*n =* 33), or affectionate/friendly (*n =* 26) responded positively to the same number of plant materials (2.5, 2.4 and 2.5 on average, respectively).

Because most (80–90%) of the cats studied were (blends of) domestic short-haired breeds, we did not study associations between breed and responsiveness to the materials tested. Also, because of the small number of cats with the same fur color and pattern, as well as the difficulties in determining these characteristics (i.e., the small number of known purebreds), we did not test for differences in responsiveness between the various fur colors and patterns.

### Overlap in responses to the various plant materials

**Fig. 5:**
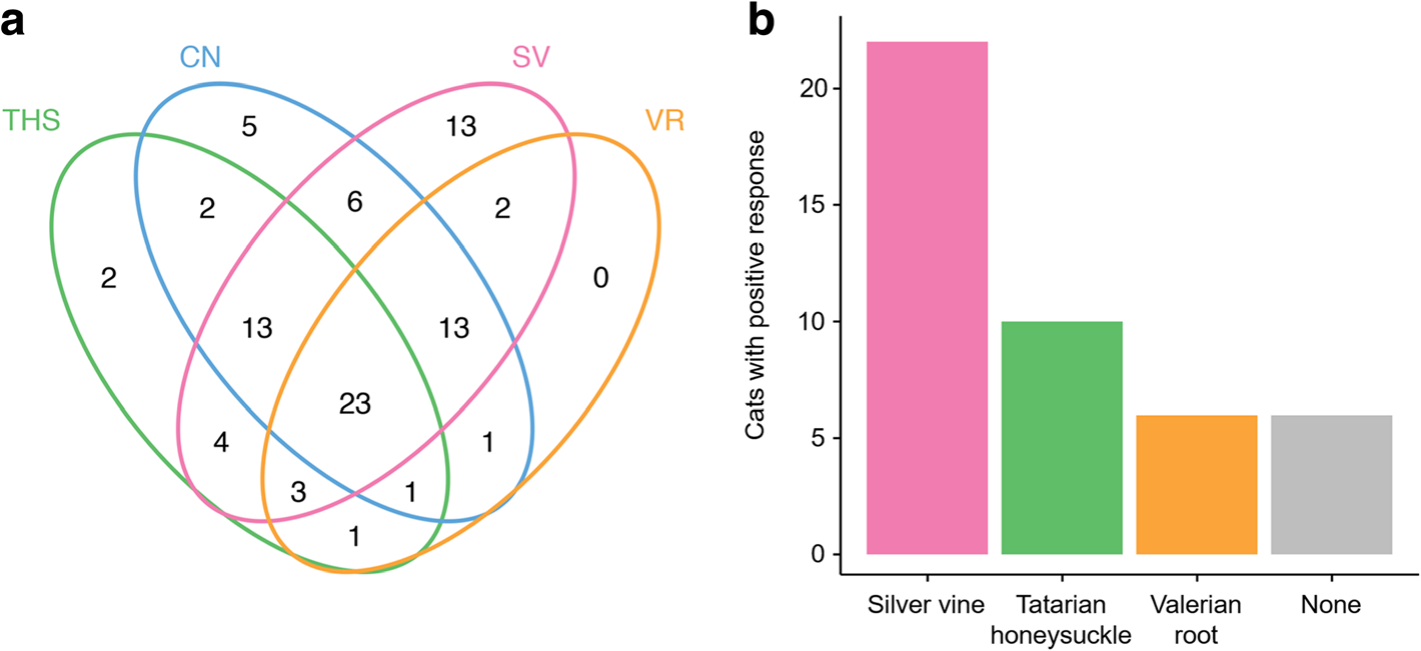
Response patterns and alternatives to catnip. **a** Venn diagram (not drawn to scale) showing the overlap in responses to catnip, silver vine, Tatarian honeysuckle and valerian root. Ninety-five of the 100 cats tested were exposed to all four plant materials, of which 89 are included in this plot. The remaining six cats did not respond to any of the plant materials and are therefore not shown in this diagram. **b** Of the 31 cats that did not respond to catnip, 22 (71%) responded positively to silver vine, 10 (32%) to Tatarian honeysuckle and 6 (19%) to valerian root

To investigate which of the plant materials elicited responses in cats not responding to catnip, we analyzed the overlap in responses to the four different plant materials. Ninety-four of the 100 cats (94%) responded to at least one of the four stimuli, whereas six cats (6%) did not respond to any of the materials. Twenty-three of the 95 cats (24%) that were exposed to all four different plant materials responded to all of them (Fig. 5a). Of the 31 cats not responding to catnip, 22 (71%) responded to silver vine, 10 (32%) to Tatarian honeysuckle, and 6 (19%) to valerian root (Fig. 5b). These results suggest that silver vine is the best alternative to catnip, also because there were only three cats out of the 95 (3%) that did respond to Tatarian honeysuckle or valerian root, but not to silver vine or catnip. Twenty of the 95 cats (21%) that were offered all four different plant materials responded to just one stimulus, the majority of which (13 out of 20; 65%) responded exclusively to silver vine, suggesting that silver vine contains chemical compounds that are either absent or present in lower concentrations in catnip, Tatarian honeysuckle and valerian root.

### Responses to various parts of the silver vine plant

**Fig. 6:**
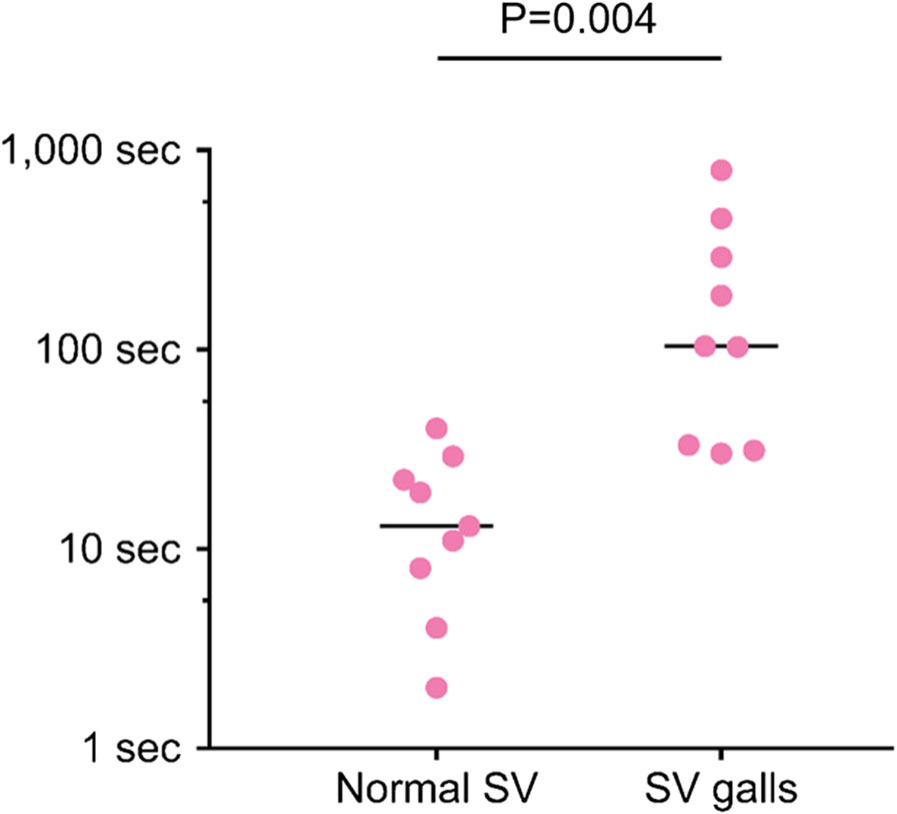
Time each cat (represented by a circle) spent with a sock filled with dried normal silver vine (SV) fruit or dried silver vine fruit galls. The socks were offered simultaneously. *Horizontal lines* represent the medians. Note that the ordinate is a logarithmic scale, with the minor ticks denoting 5, 50 and 500 s

The silver vine powder used in this study was made from dried silver vine fruit galls (Fig. 2b) (Smack and Gendai Pharmaceutical, personal communication, April 2016), caused by the gall midge *Pseudasphondylia matatabi* [24]. Matatabi fruit gall midges leave the fruit when the larvae have matured. It is speculated that cats respond more strongly to dried silver vine fruit galls than to dried normal, oval shaped fruit with a smooth surface (Fig. 2c). We tested this hypothesis by offering nine cats a sock filled with dried normal fruit and a sock filled with dried fruit galls, simultaneously (Table 2), and measured the time spent with each sock. We found that the cats spent significantly more time with the fruit galls (median facial contact time 104 s) than with the normal fruit (13 s) (*P =* 0.004, Wilcoxon matched-pairs signed rank test) (Fig. 6). Eight of the nine cats responded positively to the silver vine fruit galls. Although some time was spent with the sock containing the dried normal fruit, none of the nine cats responded positively to it.

We subsequently investigated if cats responded to silver vine woodchips made from the wood of a plant not bearing fruit galls (according to the seller), dried and fresh leaves from a plant without fruit galls, or powder made from dried normal fruit. Eight of the same nine cats (one cat had been adopted) were exposed to socks containing these materials (Table 2). None of the eight cats showed any interest in the powder made from dried normal fruit, or in the dried and fresh leaves. Although seven out of eight cats also did not respond to the silver vine wood, one cat did respond positively to this material, on four different testing occasions. However, during each of the two exposures for which we counted facial contact time, the response to the galled fruit lasted longer than to the woodchips (276 and 249 s facial contact time compared to 93 and 126 s, respectively), even though the galled fruit was offered after the cat had lost interest in the sock filled with silver vine woodchips.

### Qualitative response

The way in which cats responded to the various plant materials (Table 1) was similar to the catnip response that has been described in detail before [6, 9–11], indicating that this characteristic response is not restricted to catnip. Although we did not attempt to document the behavioral response in detail, we did observe a response to the plant materials that, to the best of our knowledge, has not been described before. In several of the responding domestic cats we observed undulation of the skin overlying the dorsal lumbosacral region of the back shortly after exposure to the plant materials. The wave-like motions only lasted a few seconds and were possibly associated with contractions of the cutaneous trunci muscles, a broad sheath of skeletal muscle that lies directly underneath the skin [25].

### Other *Felidae*: tigers and bobcats

Unlike the other big cats, tigers are known not to, or at best only mildly, respond to catnip [6, 7]. Tigers typically seem to be less interested in any form of olfactory enrichment (e.g., spices, herbs, perfumes) compared to other wild cats (L. Buckingham, Big Cat Rescue, personal observation, 2013 through 2016). Since we observed that many domestic cats that did not respond to catnip did respond to silver vine, we were interested to learn how tigers would respond to silver vine. To the knowledge of the authors, silver vine has not been tested on tigers before. Catnip and silver vine powder were offered to nine tigers. One tiger responded mildly positive (sniffing, shaking her head and licking it, for three minutes) to catnip, but the other eight tigers were indifferent to the presence of this plant material. Four of the nine tigers also were indifferent to the silver vine powder, whereas the other five responded disapprovingly to it: after sniffing the plant material the animals backed off and walked away from it. This disapproving response to silver vine was distinct from their negative response to catnip. For this reason we decided to not offer the plant materials to the tigers multiple times.

**Fig. 7:**
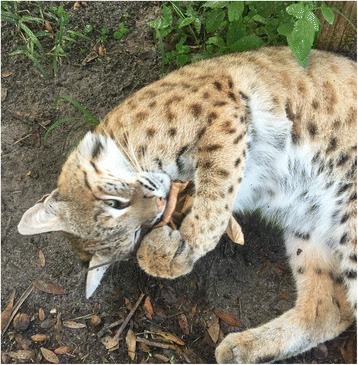
An eight year old female bobcat holding a paper bag with silver vine powder between her forelegs while she is rolling around and giving it chin and cheek rubs

In contrast to the response of the tigers, another non-domesticated member of the *Felidae*, bobcats, did respond positively to silver vine (*n* = 4) as well as to catnip (*n* = 1). This positive response was characterized by holding the paper bag containing the plant material close to their head using their forelegs (Fig. 7) while chin and cheek rubbing it, rolling over on their back from one side to the other, and drooling. This response lasted several minutes, sometimes longer than 15 min. To the knowledge of the authors, this is the first time silver vine has been tested on bobcats.

### Chemical analysis

Concentrations of *cis-trans* nepetalactone, *trans-cis* nepetalactone, actinidine, iridomyrmecin and isodihydronepetalactone in the four plant materials were determined using gas chromatography coupled with mass spectrometry. Reference compounds and reference spectra for isoiridomyrmecin (CAS Registry Number 107538-14-7), dihydronepetalactone (4581-72-0) and neonepetalactone (4581-74-2) were not (commercially) available and these compounds could therefore not be identified. The highest concentration of *cis-trans* nepetalactone was found in catnip, whereas minimal quantities were detected in the other plant materials (Table 3). Only a relatively small amount (32 μg/g) of *trans-cis* nepetalactone was found in catnip. Actinidine concentrations were highest in silver vine fruit galls, valerian root and Tatarian honeysuckle and marginal in catnip. The concentration of iridomyrmecin and isodihydronepetalactone was highest in silver vine fruit galls, while valerian root contained some iridomyrmecin and catnip some isodihydronepetalactone. In addition, relatively high concentrations of three different isodihydronepetalactone isomers (diastereomers) were identified in silver vine fruit galls.

**Table 3:** Allomone concentrations in the plant materials used in this study

	CN ^a^	SV	THS	VR
*cis-trans* Nepetalactone	1,010 ^b^	3	13	5
*trans-cis* Nepetalactone	32	0	0	0
Actinidine	11	290	108	172
Iridomyrmecin	0	167	7	31
Isodihydronepetalactone	55	164	7	0
Isodihydronepetalactone isomer 1	0	315	0	0
Isodihydronepetalactone isomer 2	33	141	0	0
Isodihydronepetalactone isomer 3	0	181	0	0

^a^ *CN* catnip, *SV* silver vine fruit galls, *THS* Tatarian honeysuckle, *VR* valerian root

^b^ Concentrations are expressed as μg per gram dried plant material

**Fig. 8:**
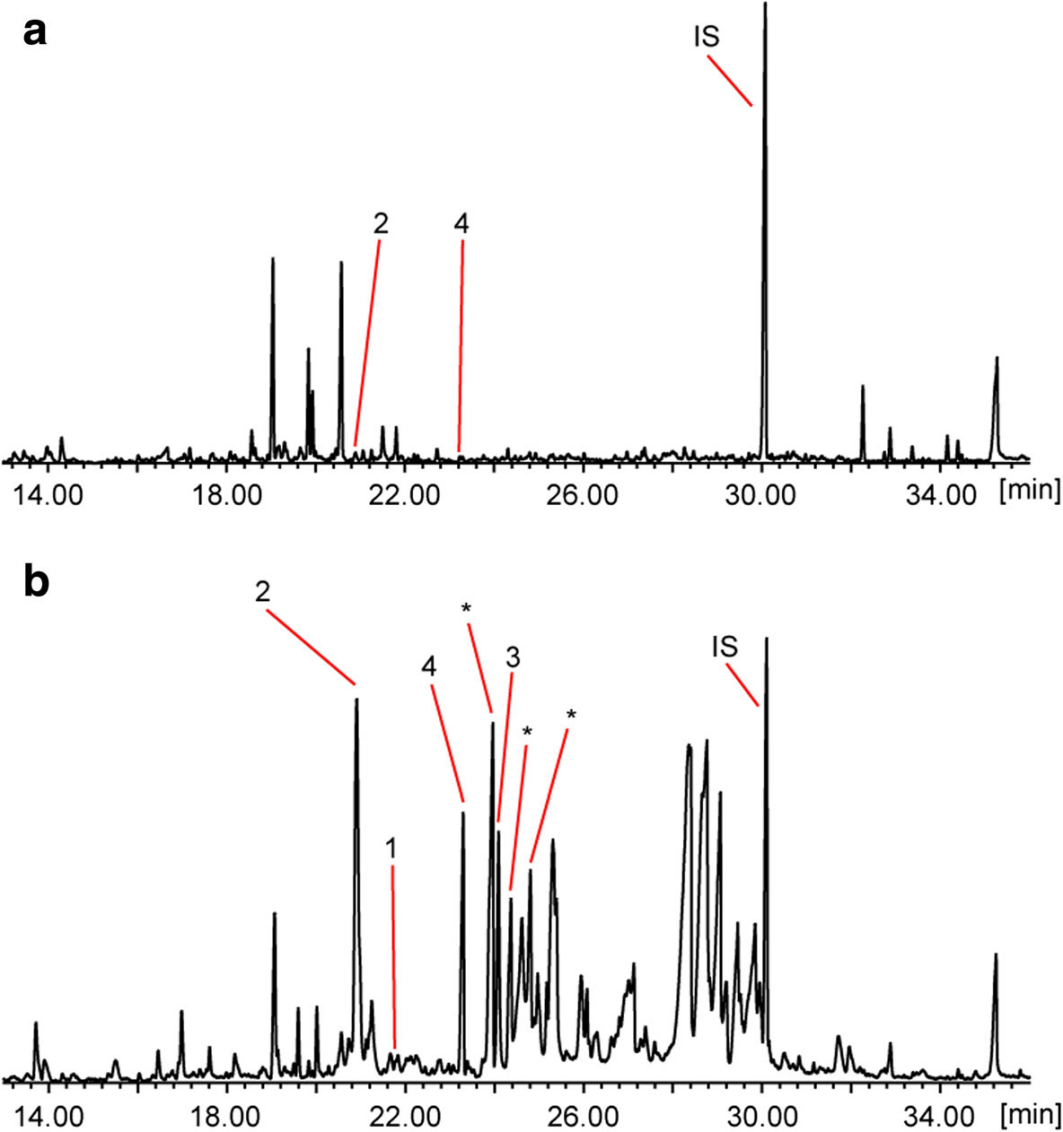
Total ion chromatograms comparing the extracts of dried normal silver vine fruit (**a**) to the extracts of dried silver vine fruit galls (**b**). The normal fruit only contained marginal levels of actinidine (2) and isodihydronepetalactone (4) compared to the fruit galls. Relatively large amounts of actinidine (2), isodihydronepetalactone (4), iridomyrmecin (3) and its isomers (*) were present in the fruit galls. Note the higher concentrations of other lactones (min. ~25 to 30) in the fruit galls. Only a small quantity of *cis-trans* nepetalactone was detected in the fruit galls. 1, *cis-trans* nepetalactone; 2, actinidine; 3, iridomyrmecin; 4, isodihydronepetalactone; * different isodihydronepetalactone isomers; IS, internal standard

We also analyzed these compounds in dried normal silver vine fruit. When comparing dried silver vine fruit galls to normal silver vine fruit, we observed higher concentrations of actinidine, iridomyrmecin and isodihydronepetalactone, as well as three isodihydronepetalactone isomers and other compounds similar in structure (lactones) in the dried fruit galls (Fig. 8). Mass spectra and retention indices of the three isodihydronepetalactone isomers are shown in Additional file 3. Concentrations of all these compounds were nearly undetectable (0 to 6 μg/g) in the dried normal silver vine fruit.

## Discussion

Olfactory stimulation by catnip is a well-known method to provide enrichment for a cat’s living environment. Although it has been long known that not all cats respond to catnip, only limited data are available about plants that may be used as an alternative. This study was designed to compare the responsiveness of cats to catnip, silver vine, Tatarian honeysuckle and valerian root. The sample size (*n =* 100) and the comparison of responses to four different plant materials make this study the most comprehensive on this subject thus far. In addition, this is the first study to document responses of domestic cats to silver vine fruit galls, Tatarian honeysuckle and valerian root, and those of tigers and bobcats to catnip and silver vine.

Nearly all domestic cats responded to at least one of the plant materials tested, with the largest numbers of cats responding to silver vine (79%) and catnip (68%). Our results indicate that cats predominantly responded to the powder of silver vine fruit galls, and much less frequently to the wood of the silver vine plant. In contrast, Katahira and Iwai [21] found that 28 out of 31 cats (90%) responded positively to a dried silver vine branch. This discrepancy may be explained by the methodology of their study. Experiments were performed on laboratory cats housed in small cages (39 × 51 × 33 cm) that were offered a dried branch of silver vine. Approaching and trying to bite the branch within three minutes was considered a positive response, and no negative controls were used in their study.

**Fig. 9:**
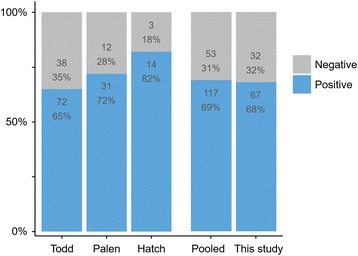
The absolute number and percentage of cats that responded positively to catnip in three previous studies [9–11] (*left*). The pooled results from these three studies are similar to our findings (*right*)

The percentage of non-responders to catnip in our study is in line with findings from previous studies: 35% [9], 28% [10] and 18% [11]. Pooling the data from these studies gives a total of 53 non-responders out of 170 cats (31%), which is very similar to the 32 out of 99 cats (32%) that did not respond to catnip in our study (Fig. 9). Also in line with our observations, a similar percentage of cats with mild or partial responses to catnip was reported by Palen and Goddard [10] (8 out of 43 or 19% compared to 16 out of 99 or 16% in our study).

Our observation that tigers did not respond to catnip agrees with results of previous studies. Todd found that 15 of the 23 tigers (65%) tested did not respond at all, six (26%) only sniffed, licked and shook their head, and two tigers (9%) showed a partial response including chin and cheek rubs [6]. None of the tigers rolled over. Hill et al. found that three out of four tigers did not respond to catnip, whereas one showed a mild or partial response [7]. Results from these studies also indicated that all other members of the genus *Panthera* (lions, jaguars, leopards and snow leopards) tested, did respond positively to catnip. Todd and Hill et al. also tested catnip on bobcats [6, 7] and their findings were contradictory, possibly because only two bobcats were tested in each study.

Although data specific for cats are not available, all four plant species used in this study are generally considered safe and not toxic or addictive for cats and humans. Cats have been exposed to silver vine and catnip, in a variety of different forms, for many years. We could only find one anecdote related to the potential addictive properties of silver vine [26]. In the 1970s, the smell of an unspecified form and concentration of silver vine was reportedly preferred by large cats (species not specified by the author) kept in cages in a zoo in Japan over food and sexual intercourse. However, their response to the silver vine was one of excitement and joy; no data were presented supporting any negative health effects. The scientific value of their claim that silver vine is addictive recently has been disputed by others [27], and there is currently no scientific data supporting any addiction potential, nor are there any other negative anecdotes known to the authors, despite the widespread use of silver vine in Japan. A recent literature review on the use of silver vine concluded that its enrichment potential has been largely overlooked, and the authors encouraged further investigation of feline behavioral responses to silver vine [27]. The Cat House Inc. has sold thousands of pieces of Tatarian honeysuckle since 1991, and has never received a report of adverse reactions (J. Wegiel, personal communication, April 2016). None of the four plants used in this study are listed as poisonous or toxic by the Animal Poison Control Center (SafetyCall International, Bloomington, MN, USA) or the American Society for the Prevention of Cruelty to Animals (New York, NY, USA). All plant materials used in this study are commercially available, and are sold as (part of) toys or enrichment products specifically for cats. Finally, inquiry to the Pet Poison Helpline (SafetyCall International) (22 June 2016; case number 1849511) taught us that valerian root is generally considered safe, and that no toxicity has been reported from exposure to silver vine or Tatarian honeysuckle wood. The berries of Tatarian honeysuckle (not used in this study and not believed to have a stimulating effect on cats) may cause mild gastrointestinal irritation because of the presence of terpenoids.

The results of our chemical analysis suggest there are significant differences in the concentrations of compounds known or claimed to have a stimulating effect on cats between the various plant materials tested. However, interpretation of Table 3 is somewhat hampered by lack of knowledge of the limit of detection for these compounds for cats, and if there is a dose-response relationship. Our findings suggest that *cis-trans* nepetalactone is the dominant and possibly the only compound in catnip to which cats respond. The higher concentrations of actinidine in silver vine, Tatarian honeysuckle and valerian root may explain, in part, the responsiveness of cats that did not respond positively to catnip to these plant materials. In addition, cats that only responded to silver vine may specifically detect iridomyrmecin and isodihydronepetalactone in silver vine fruit galls (Table 3). Indeed, 25% (19/77) of the cats that responded to silver vine did not respond to Tatarian honeysuckle or valerian root, despite similar concentrations of actinidine in these plant materials. In normal silver vine fruit, we detected actinidine at very low concentrations (6 μg/g) compared to the fruit galls (Fig. 8), Tatarian honeysuckle, and valerian root. Sakan et al. also detected actinidine in leaves of the silver vine plant [17], but did not report the concentration they detected. Like normal silver vine fruit, leaves did not elicit a response in any of the cats we tested, so it is likely that the concentration of actinidine in silver vine leaves is also very low. While actinidine has previously been identified in silver vine and valerian root [17–20], this is, to the knowledge of the authors, the first study demonstrating that Tatarian honeysuckle contains actinidine, and that the amount is comparable to concentrations found in silver vine fruit galls and valerian root (Table 3). The low levels of *cis-trans* and *trans-cis* nepetalactone, iridomyrmecin and isodihydronepetalactone in Tatarian honeysuckle suggest that actinidine may be the only compound in the wood that cats respond to. Indeed, we found large overlaps between the plant materials that contained high concentrations of actinidine: 93% (41/44) and 88% (43/49) of the cats that responded positively to valerian root or Tatarian honeysuckle also responded to silver vine (Fig. 5a). However, because of technical limitations, we were unable to identify all of the known or claimed active compounds, which leaves room for the possibility that other compounds are involved as well. This possibility is further supported by the observation that despite similar concentrations of all the measured compounds in Tatarian honeysuckle and valerian root, only 28 of the 65 cats (43%) that responded positively to Tatarian honeysuckle or valerian root responded positively to both these plant materials. The popularity of silver vine fruit galls among cats may be explained by the relatively high concentrations of several compounds: actinidine, iridomyrmecin, isodihydronepetalactone and its isomers, although it is unknown if the latter have a stimulating effect on cats. The observation that fewer cats responded positively to silver vine wood than to silver vine fruit galls suggests that lower concentrations of these compounds are present in the wood and that the concentration of these chemicals in the plant material as well as the detection threshold of the cat contribute to the outcome of the cat’s response.

Evidence that certain compounds have an apparently euphoric effect on cats is scarce. In the early 1940s, McElvain and colleagues performed a bioassay using seven adult lions to demonstrate that nepetalactone is the active compound in catnip, but they did not discriminate between the *cis-trans* and *trans-cis* form of nepetalactone [16]. Results from subsequent studies that investigated which of the two nepetalactone isomers causes the catnip response suggest both isomers do, but the design of these studies and the reporting thereof were of poor quality [22, 23]. While Sakan and colleagues claimed that certain compounds in silver vine have cat-attracting or stimulating properties, evidence was not presented [17–19]. The limited information available about bioassays performed by Sakan and colleagues was written in Japanese [18]. They stated: “We mainly used cats and a compound was considered active when one or more of the following were observed: express interest in the sample, lick the sample, salivate, exhibit the Flehmen response, rub their neck against something, rub their back against the ground and move around, become entranced, or fall asleep. Actinidine and matatabilactone (comment by the authors: at the time matatabilactone was believed to be a mixture of iridomyrmecin and isoiridomyrmecin [19]) showed strong activity in lions, tigers and panthers, and actinidine even showed some activity in dogs.” Other than this, no data were provided to support their claims. Well-controlled studies in which cats are exposed to isolated, pure compounds and scored according to relevant and well-defined behavioral characteristics are required to conclusively establish which compounds cause the apparently euphoric response of cats to the various plant materials.

The precise mechanism by which allomone production in silver vine and other plants is stimulated and regulated is unclear. It is known that some gall-inducing insects promote growth of plant tissue to help provide them with food and shelter [28]. It seems that in response to the invasion of the fruit by the larvae, silver vine plants produce and secrete volatile compounds trying to repel the matatabi fruit gall midge. Alternatively, these compounds could function by attracting midge predators or parasitoids. Because nepetalactone has been found to be an insect repellant [29, 30] and all the cat-stimulating compounds are similar in structure (Fig. 1), the former may be more plausible. Although few in number, we did see some cats respond positively to silver vine wood, whereas no cats responded to silver vine leaves or normal silver vine fruit. We did not perform chemical analysis of silver vine wood tissue. Actinidine, iridomyrmecin, isoiridomyrmecin, dihydronepetalactone, isodihydronepetalactone and neonepetalactone were first identified in silver vine leaves and silver vine fruit galls [17, 19]. It was not specified if these leaves were from silver vine plants with or without fruit galls. In addition, it is unknown if the plant’s response to the local attack by the matatabi fruit gall midge larvae is local or systemic, and thus if allomone concentrations can increase in leaves and wood of the plant. It would be interesting to learn whether cats respond differently to silver vine wood from plants bearing galled fruit as compared to wood from vines without fruit galls.

Each of the four plant materials tested in this study can be a valuable addition to a cat’s environment, whether indoor or outdoor, but some come with a few disadvantages. Many cats respond to catnip, which is inexpensive and easy to obtain or grow. Silver vine, although perhaps even more popular among cats than catnip, is more expensive and difficult to obtain; it is predominantly available online and comes from East Asia. Producing silver vine fruit galls in areas outside of the natural habitat of silver vine is practically impossible because it would require the presence of *Pseudasphondylia matatabi*. The life span of the adult fly is one to two days. Females of *P. matatabi* lay their eggs in the flower buds of silver vine in May. The larvae spend the summer in the fruit galls, but silver vine is not flowering when the flies emerge from the fruit galls in autumn. Because of their short life span, the gall midge needs to use a different host plant during the winter season to survive [31]. To this day, this host has not been identified (Dr. J. Yukawa, Entomological Laboratory, Kyushu University, Fukuoka, Japan, personal communication, June 2016). Although inexpensive and easy to obtain, valerian root comes with a strong smell that is not appreciated by everybody. Tatarian honeysuckle wood may be the most difficult plant material to obtain, but the wood comes in many different sizes, and will last a lifetime.

The various plant materials tested may be used to increase playtime of cats, which is especially valuable for less active, obese or under-stimulated cats. They also can be given to cats for distraction when left home alone for a long time. In animal shelters, these plant materials could be used by staff or volunteers to help socialize cats or to increase the chance of rehoming more timid adult cats. Indeed, playfulness of a cat was identified as one of the most important selection criteria used by potential adopters when choosing a cat [32]. We have observed several fearful, withdrawn cats, brought into the sanctuary days or weeks before we tested them, that were attracted by the plant materials and responded seemingly euphorically after encountering them. These cats would normally hide when staff or volunteers were present. This suggests that trap-neuter-return programs also may benefit from using these plant materials. Indeed, a previous study already suggested that catnip may be effective for luring feral cats [33]. The plant materials also might be used for training purposes, where exposure to these stimuli may be used as a reward instead of food, which is most often used.

Recent studies, including a systematic review, have demonstrated the lack of evidence that synthetic feline pheromones (e.g., Feliway) can relieve stress in cats [34, 35]. It would be worthwhile to investigate if the plant materials studied here can be used to alleviate distress in cats, for example during medical procedures, transportation or boarding. Before these plant materials can be used for anything other than enrichment, it is critical that multiple well-controlled, independent studies with sufficient sample size have demonstrated their effectiveness.

## Conclusion

Olfactory enrichment using silver vine, Tatarian honeysuckle or valerian root may, similar to catnip, be an effective means to improve the quality of life for cats. Nearly all cats responded positively to at least one of these plants. For cats that do not respond to catnip, these other plants are good alternatives. Because veterinarians and their support staff are the primary educators of cat owners, it is of great importance that they become aware of the existence of these plant materials and the potential response of cats to them. Future studies will be needed to evaluate if these plant materials can be used to improve the quality of life for confined cats, reduce distress, for training, socializing or in trap-neuter-return programs.

## Additional files

Additional file 1: Wash-out period.tif. Schematic representation (time-line) of the results from our preliminary experiment to establish the wash-out period. Four cats where offered a sock with 1 g silver vine (SV) powder (Smack) and an empty control sock. The sock with SV was removed when an intense (positive) response was observed and was replaced with the negative control sock. The positive response ceased immediately or near immediately (seconds) after the plant material was removed from the cat. The sock with SV was reintroduced to confirm that this loss of the positive response was not because of fatigue, distraction or loss of interest. Indeed, on most occasions a positive response was observed again instantly after exposure to the reintroduced SV sock. These results suggest that the risk of a carry-over effect (behavior associated with a positive response) from one plant material to another is minimal when at least 5 min transpire between the exposures. (TIF 152 kb)
Additional file 2: Raw data.xlsx. Microsoft Excel 2013 file containing raw data (among others gender, age and responsiveness scores) for the domestic cats as well as for the tigers and bobcats. (XLSX 17 kb)
Additional file 3: Isodihydronepetalactone isomers mass spectra.tif. Mass spectra and retention indices of three isodihydronepetalactone isomers that were detected in silver vine fruit galls, but not in normal silver vine fruit. RI, retention index. (TIF 468 kb)
