# Supplementary figures and images for: Responsiveness of cats (Felidae) to silver vine (Actinidia polygama), Tatarian honeysuckle (Lonicera tatarica), valerian (Valeriana officinalis) and catnip (Nepeta cataria)
Source: BMC Vet Res. 2017 Mar 16;13:70. doi: 10.1186/s12917-017-0987-6 (PMC5356310; doi:10.1186/s12917-017-0987-6)

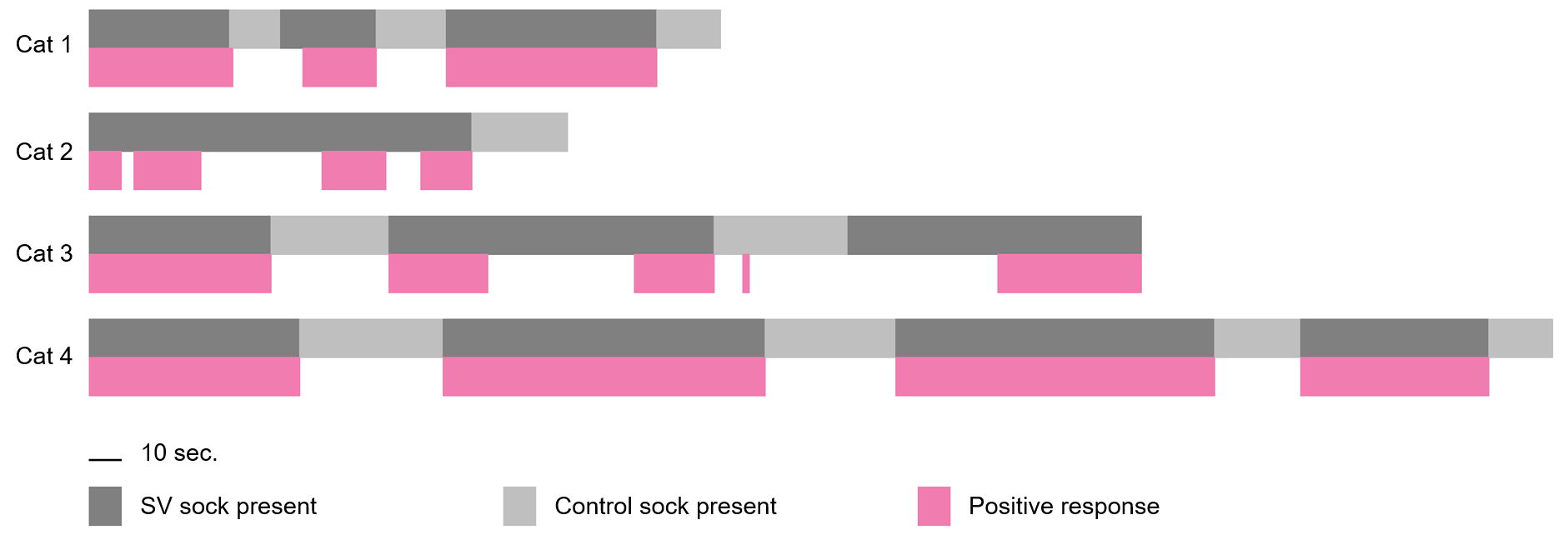

Supplement: Additional file 1: — Wash-out period.tif. Schematic representation (time-line) of the results from our preliminary experiment to establish the wash-out period. Four cats where offered a sock with 1 g silver vine (SV) powder (Smack) and an empty control sock. The sock with SV was removed when an intense (positive) response was observed and was replaced with the negative control sock. The positive response ceased immediately or near immediately (seconds) after the plant material was removed from the cat. The sock with SV was reintroduced to confirm that this loss of the positive response was not because of fatigue, distraction or loss of interest. Indeed, on most occasions a positive response was observed again instantly after exposure to the reintroduced SV sock. These results suggest that the risk of a carry-over effect (behavior associated with a positive response) from one plant material to another is minimal when at least 5 min transpire between the exposures. (TIF 152 kb) [file 12917_2017_987_MOESM1_ESM.tif]

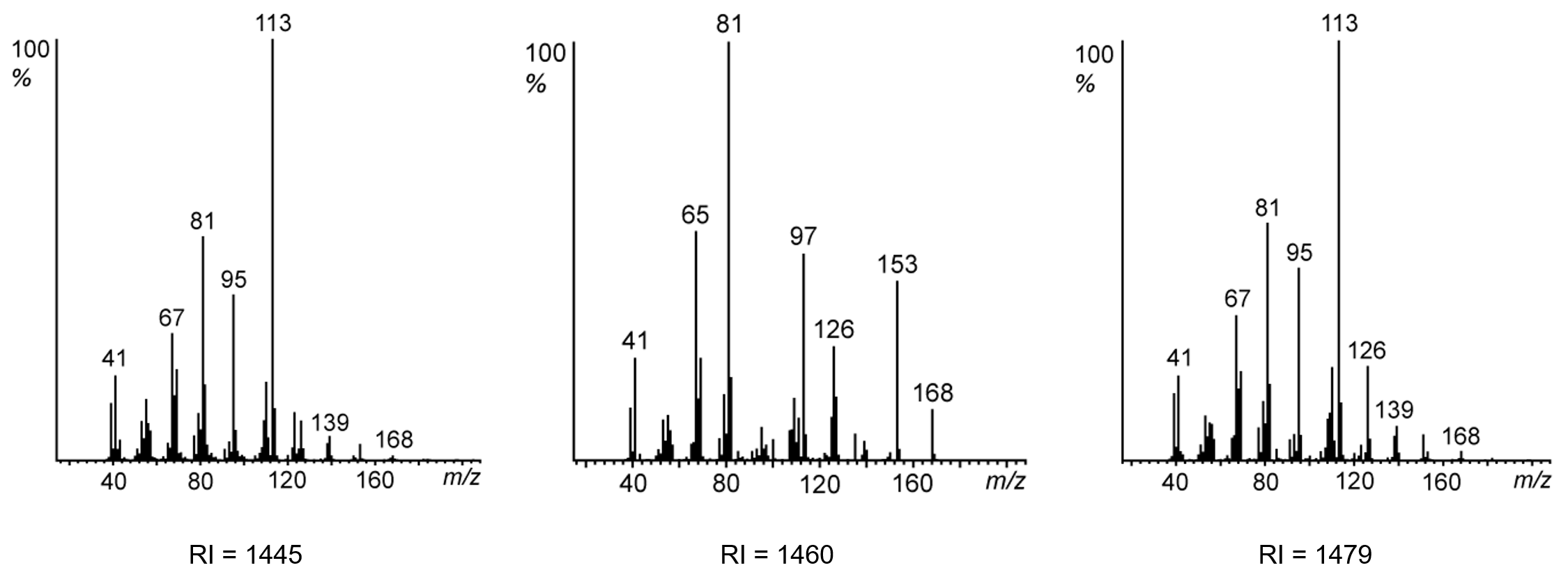

Supplement: Additional file 3: — Isodihydronepetalactone isomers mass spectra.tif. Mass spectra and retention indices of three isodihydronepetalactone isomers that were detected in silver vine fruit galls, but not in normal silver vine fruit. RI, retention index. (TIF 468 kb) [file 12917_2017_987_MOESM3_ESM.tif]
